# Predicting p***K***_***a***_ values from EEM atomic charges

**DOI:** 10.1186/1758-2946-5-18

**Published:** 2013-04-10

**Authors:** Radka Svobodová Vařeková, Stanislav Geidl, Crina-Maria Ionescu, Ondřej Skřehota, Tomáš Bouchal, David Sehnal, Ruben Abagyan, Jaroslav Koča

**Affiliations:** 1 National Centre for Biomolecular Research, Faculty of Science and CEITEC - Central European Institute of Technology, Masaryk University Brno, Kamenice 5, 625 00 Brno-Bohunice, Czech Republic; 2 Skaggs School of Pharmacy and Pharmaceutical Sciences, University of California, 9500 Gilman Drive, MC 0657 San Diego, USA

**Keywords:** Dissociation constant, Quantitative structure-property relationship, QSPR, Partial atomic charges, Electronegativity equalization method, EEM, Quantum mechanics, QM

## Abstract

The acid dissociation constant p *K*_*a*_is a very important molecular property, and there is a strong interest in the development of reliable and fast methods for p *K*_*a*_prediction. We have evaluated the p *K*_*a*_prediction capabilities of QSPR models based on empirical atomic charges calculated by the Electronegativity Equalization Method (EEM). Specifically, we collected 18 EEM parameter sets created for 8 different quantum mechanical (QM) charge calculation schemes. Afterwards, we prepared a training set of 74 substituted phenols. Additionally, for each molecule we generated its dissociated form by removing the phenolic hydrogen. For all the molecules in the training set, we then calculated EEM charges using the 18 parameter sets, and the QM charges using the 8 above mentioned charge calculation schemes. For each type of QM and EEM charges, we created one QSPR model employing charges from the non-dissociated molecules (three descriptor QSPR models), and one QSPR model based on charges from both dissociated and non-dissociated molecules (QSPR models with five descriptors). Afterwards, we calculated the quality criteria and evaluated all the QSPR models obtained. We found that QSPR models employing the EEM charges proved as a good approach for the prediction of p *K*_*a*_(63% of these models had *R*^2^ > 0.9, while the best had *R*^2^ = 0.924). As expected, QM QSPR models provided more accurate p *K*_*a*_predictions than the EEM QSPR models but the differences were not significant. Furthermore, a big advantage of the EEM QSPR models is that their descriptors (i.e., EEM atomic charges) can be calculated markedly faster than the QM charge descriptors. Moreover, we found that the EEM QSPR models are not so strongly influenced by the selection of the charge calculation approach as the QM QSPR models. The robustness of the EEM QSPR models was subsequently confirmed by cross-validation. The applicability of EEM QSPR models for other chemical classes was illustrated by a case study focused on carboxylic acids. In summary, EEM QSPR models constitute a fast and accurate p *K*_*a*_prediction approach that can be used in virtual screening.

**Electronic supplementary material:**

The online version of this article (doi:10.1186/1758-2946-5-18) contains supplementary material, which is available to authorized users.

## Background

The acid dissociation constant p *K*_*a*_is an important molecular property, and its values are of interest in pharmaceutical, chemical, biological and environmental research. The p *K*_*a*_values have found application in many areas, such as the evaluation and optimization of candidate drug molecules [1–3], ADME profiling [4, 5], pharmacokinetics [6], understanding of protein-ligand interactions [7, 8], etc.. Moreover, the key physicochemical properties like lipophilicity, solubility, and permeability are all p *K*_*a*_dependent. For these reasons, p *K*_*a*_values are important for virtual screening. Therefore, both the research community and pharmaceutical companies are interested in the development of reliable and above all fast methods for p *K*_*a*_prediction.

Several approaches for p *K*_*a*_prediction have been developed [8–11], namely LFER (Linear Free Energy Relationships) methods [12, 13], database methods, decision tree methods [14], ab initio quantum mechanical calculations [15, 16], ANN (artificial neural networks) methods [17] or QSPR (quantitative structure-property relationship) modelling [18–20]. However, p *K*_*a*_values remain one of the most challenging physicochemical properties to predict. A promising approach for p *K*_*a*_prediction is to use QSPR models which employ partial atomic charges as descriptors [21–24].

The partial atomic charges cannot be determined experimentally and they are also not quantum mechanical observables. For this reason, the rules for determining partial atomic charges depend on their application (e.g. molecular mechanics energy, p *K*_*a*_etc.), and many different methods have been developed for their calculation. The charge calculation methods can be divided into two main groups, namely quantum mechanical (QM) approaches and empirical approaches.

The quantum mechanical approaches first calculate a molecular wave function by a combination of some theory level (e.g., HF, B3LYP, MP2) and basis set (e.g., STO-3G, 6–31G*), and then partition this wave function among the atoms (i.e., the assignment of a specific part of the molecular electron density to each atom). This partitioning can be done using an orbital-based population analysis, such as MPA (Mulliken population analysis) [25, 26], Löwdin population analysis [27] or NPA (natural population analysis) [28]. Other partitioning approaches are based on a wave-function-dependent physical observable. Such approaches are, for example, AIM (atoms in molecules) [29], Hirshfeld population analysis [30] and electrostatic potential fitting methods like CHELPG [31] or MK (Merz-Singh-Kollman) [32]. Another partitioning method is the mapping of QM atomic charges to reproduce charge-dependent observables (e.g., CM1, CM2, CM3 and CM4) [33].

Empirical approaches determine partial atomic charges without calculating a quantum mechanical wave function for the given molecule. Therefore they are markedly faster than QM approaches. One of the first empirical approaches developed, CHARGE [34], performs a breakdown of the charge transmission by polar atoms into one-bond, two-bond, and three-bond additive contributions. Most of the other empirical approaches have been derived on the basis of the electronegativity equalization principle. One group of these empirical approaches invoke the Laplacian matrix formalism, and result in a redistribution of electronegativity. Such methods are PEOE (partial equalization of orbital electronegativity) [35], GDAC (geometry-dependent atomic charge) [36], KCM (Kirchhoff charge model) [37], DENR (dynamic electronegativity relaxation) [38] or TSEF (topologically symmetric energy function) [38]. The second group of approaches use full equalization of orbital electronegativity, and such approaches are, for example, EEM (electronegativity equalization method) [39], QEq (charge equilibration) [40] or SQE (split charge equilibration) [41]. The empirical atomic charge calculation approaches can also be divided into ’topological’ and ’geometrical’. Topological charges are calculated using the 2D structure of the molecule, and they are conformationally independent (i.e., CHARGE, PEOE, KCM, DENR, and TSEF). Geometrical charges are computed from the 3D structure of the molecule and they consider the influence of conformation (i.e., GDAC, EEM, Qeq, and SQE).

The prediction of p *K*_*a*_using QSPR models which employ QM atomic charges was described in several studies [21–24], which have analyzed the precision of this approach and compared the quality of QSPR models based on different QM charge calculation schemes. All these studies show that QM charges are successful descriptors for p *K*_*a*_prediction, as the QSPR models based on QM atomic charges are able to calculate p *K*_*a*_with high accuracy. The weak point of QM charges is that their calculation is very slow, as the computational complexity is at least *θ* (*E*^4^), where *E* is the number of electrons in the molecule. Therefore, p *K*_*a*_prediction by QSPR models based on QM charges cannot be applied in virtual screening, as it is not feasible to compute QM atomic charges for hundreds of thousands of compounds in a reasonable time. This issue can be avoided if empirical charges are used instead of QM charges. A few studies were published, which give QSPR models for predicting p *K*_*a*_using topological empirical charges as descriptors (specifically PEOE charges) [22, 42, 43]. But these models provided relatively weak predictions.

The geometrical charges seem to be more promissing descriptors, because they are able to take into consideration the influence of the molecule’s conformation on the atomic charges. The conformation of the atoms surrounding the dissociating hydrogens strongly influences the dissociation process, and also the atomic charges.

The EEM method is a geometrical empirical charge calculation approach which can be useful for p *K*_*a*_prediction by QSPR. This approach calculates charges using the following equation system:

1 $$\left(\begin{array}{ccccc} B_{1} & \frac{\kappa }{R_{1,2}} & \ldots & \frac{\kappa }{R_{1,N}} & -1\\ \frac{\kappa }{R_{2,1}} & B_{2} & \ldots & \frac{\kappa }{R_{2,N}} & -1\\ \vdots & \vdots & \ddots & \vdots & \vdots \\ \frac{\kappa }{R_{N,1}} & \frac{\kappa }{R_{N,2}} & \ldots & B_{N} & -1\\ 1 & 1 & \ldots & 1 & 0 \end{array}\right)\;\left(\begin{array}{c} q_{1}\\ q_{2}\\ \vdots \\ q_{N}\\ \bar{\chi } \end{array}\right)=\left(\begin{array}{c} -A_{1}\\ -A_{2}\\ \vdots \\ -A_{N}\\ Q \end{array}\right)$$

where *q*_*i*_is the charge of atom *i*; *R*_*i*,*j*_is the distance between atoms *i* and *j*; *Q* is the total charge of the molecule; *N* is the number of atoms in the molecule; $\bar{\chi }$ is the molecular electronegativity, and *A*_*i*_, *B*_*i*_and *κ* are empirical parameters. These parameters are obtained by a parameterization process, which uses QM atomic charges to calculate a set of parameters for which EEM best reproduces these QM charges. EEM is very popular, and despite the fact that it was developed more than twenty years ago, new parameterizations [39, 44–50] and modifications [47, 51, 52] of EEM are still under development. Its accuracy is comparable to the QM charge calculation approach for which it was parameterized. Additionally, EEM is very fast, as its computational complexity is *θ* (*N*^3^), where *N* is the number of atoms in the molecule.

Therefore, in the present study, we focus on p *K*_*a*_prediction using QSPR models which employ EEM charges. Specifically, we created and evaluated QSPR models based on EEM charges computed using 18 EEM parameter sets. We also compared these QSPR models with corresponding QSPR models which employ QM charges computed by the same charge calculation schemes used for EEM parameterization.

## Methods

### EEM parameter sets

In our study, we used all EEM parameters published till now. Specifically, we found 18 different EEM parameters sets, published in 8 different articles [39, 44–50]. The parameters cover two QM theory levels (HF and B3LYP), two basis sets (STO-3G and 6–31G*) and six population analyses (MPA, NPA, Hirshfeld, MK, CHELPG, AIM). Unfortunately, only some combinations of QM theory levels, basis sets and population analyses are available. On the other hand, more parameter sets were published for some combinations (i.e., 6 parameter sets for HF/STO-3G/MPA). All the parameter sets include parameters for C, O, N and H. Some sets include also parameters for S, P, halogens and metals. Most of the sets do not include parameters for C and N bonded by triple bond. Summary information about all these parameter sets is given in Table 1.

**Table 1:** **Summary information about the EEM parameter sets used in the present study**

QM theory level	PA	EEM parameter	Published by	Year of	Elements included
+ basis set		set name		publication	
HF/STO-3G	MPA	Svob2007_cbeg2	Svobodova et al. [44]	2007	C, O, N, H, S
		Svob2007_cmet2	Svobodova et al. [44]	2007	C, O, N, H, S, Fe, Zn
		Svob2007_chal2	Svobodova et al. [44]	2007	C, O, N, H, S, Br, Cl, F, I
		Svob2007_hm2	Svobodova et al. [44]	2007	C, O, N, H, S, F, Cl, Br, I, Fe, Zn
		Baek1991	Baekelandt et al. [45]	1991	C, O, N, H, P, Al, Si
		Mort1986	Mortier et al. [39]	1986	C, O, N, H
HF/6–31G*	MK	Jir2008_hf	Jirouskova et al. [46]	2008	C, O, N, H, S, F, Cl, Br, I, Zn
B3LYP/6–31G*	MPA	Chaves2006	Chaves et al. [47]	2006	C, O, N, H, F
		Bult2002_mul	Bultinck et al. [48]	2002	C, O, N, H, F
	NPA	Ouy2009	Ouyang et al. [49]	2009	C, O, N, H, F
		Ouy2009_elem	Ouyang et al. [49]	2009	C, O, N, H, F
		Ouy2009_elemF	Ouyang et al. [49]	2009	C, O, N, H, F
		Bult2002_npa	Bultinck et al. [48]	2002	C, O, N, H, F
	Hir.	Bult2002_hir	Bultinck et al. [48]	2002	C, O, N, H, F
	MK	Jir2008_mk	Jirouskova et al. [46]	2008	C, O, N, H, S, F, Cl, Br, I, Zn
		Bult2002_mk	Bultinck et al. [48]	2002	C, O, N, H, F
	CHELPG	Bult2002_che	Bultinck et al. [48]	2002	C, O, N, H, F
	AIM	Bult2004_aim	Bultinck et al. [50]	2004	C, O, N, H, F

### EEM charge calculation

The EEM charges were calculated by the program EEM SOLVER [53] using each of the 18 EEM parameter sets.

### QM charge calculation

We calculated QM atomic charges for all the combinations of QM theory level, basis set and population analysis for which we have EEM parameters (see Table 1). Specifically, atomic charges were calculated for these eight QM approaches: HF/STO-3G/MPA, HF/6–31G*/MK, and B3LYP/6–31G* with MPA, NPA, Hirshfeld, MK, CHELPG and AIM). The QM charge calculations were carried out using Gaussian09 [54]. In the case of AIM population analysis, the output from Gaussian09 was further processed by the software package AIMAll [55].

### Data set for phenols

There are two main ways to create a QSPR model for a feature to be predicted. The first is to create as general a model as possible, with the risk that the accuracy of such a model may not be high. The second approach is to develop more models, each of them being dedicated to a certain class of compounds. Here we took the second approach, following a similar methodology as in previous studies [21–24]. Specifically, we focus on substituted phenols, because they are the most common test set molecules employed in the evaluation of novel p *K*_*a*_prediction approaches [21–24, 56–58]. Our data set contains the 3D structures of 74 distinct phenol molecules. This data set is of high structural diversity and it covers molecules with p *K*_*a*_values from 0.38 to 11.1. The molecules were obtained from the NCI Open Database Compounds [59] and their 3D structures were generated by CORINA 2.6 [60], without any further geometry optimization. Our data set is a subset of the phenol data set used in our previous work related to p *K*_*a*_prediction from QM atomic charges [24]. The subset is made up of phenols which contain only C, O, N and H, and none of the molecules contain triple bonds. This limitation is necessary, because the EEM parameters of all 18 studied EEM parameter sets are available only for such molecules (see Table 1). For each phenol molecule from our data set, we also prepared the structure of the dissociated form, where the hydrogen is missing from the phenolic OH group. This dissociated molecule was created by removing the hydrogen from the original structure without subsequent geometry optimization. The list of the molecules, including their names, NCS numbers, CAS numbers and experimental p *K*_*a*_values, can be found in the (Additional file 1: Table S1a). The SDF files with the 3D structures of molecules and their dissociated forms are also in the (Additional file 2: Molecules).

### Data set for carboxylic acids

An aspect which is very important for the applicability of the p *K*_*a*_prediction approach is its transferability to other chemical classes. In this work, we provide a case study showing the performance of the approach on carboxylic acids, which are also very common testing molecules for p *K*_*a*_prediction approaches [19–21, 43]. The data set contains 71 distinct molecules of carboxylic acids and their dissociated forms. The 3D structures of these molecules were obtained in the same way as for the phenols. The list of the molecules, including their names, NCS numbers, CAS numbers and experimental p *K*_*a*_values can be found in the (Additional file 3: Table S1b). The SDF files with the 3D structures of the molecules and their dissociated forms are also included in the (Additional file 2: Molecules).

### p ***K***_***a***_values

The experimental p *K*_*a*_values were taken from the Physprop database [61].

### Descriptors and QSPR models for phenols

Our descriptors were atomic charges. We analyzed two types of QSPR models, namely QSPR models with three descriptors (3d QSPR models) and QSPR models with five descriptors (5d QSPR models).

The 3d QSPR models used those descriptors which proved to be the most relevant for p *K*_*a*_prediction in our previous study [24]. Therefore these descriptors were the atomic charge of the hydrogen atom from the phenolic OH group (*q*_*H*_), the charge on the oxygen atom from the phenolic OH group (*q*_*O*_), and the charge on the carbon atom binding the phenolic OH group (*q*_*C*1_). These descriptors were used to establish the QSPR models by the general equation:

2 $$pK_{a}=p_{H}\cdot q_{H}+p_{O}\cdot q_{O}+p_{C1}\cdot q_{C1}+p$$

where *p*_*H*_, *p*_*O*_, *p*_*C*1_ and *p* are parameters of the QSPR model (i.e., constants derived by multiple linear regression). The 5d QSPR models employ the above mentioned descriptors *q*_*H*_, *q*_*O*_and *q*_*C*1_ and additionally also the charge on the phenoxide O^-^ from the dissociated molecule (*q*_*OD*_), and the charge on the carbon atom binding this oxygen (*q*_*C*1*D*_). Using the charges from the dissociated molecules for p *K*_*a*_prediction was inspired by the work of Dixon et al. [19]. The equation of the 5d QSPR models is therefore:

3 $$\;pK_{a}\;=\;p_{H}^{\prime }\cdot q_{H}+p_{O}^{\prime }\cdot q_{O}+p_{C1}^{\prime }\cdot q_{C1}+p_{\text{OD}}^{\prime }\cdot q_{\text{OD}}+p_{C1D}^{\prime }\cdot q_{C1D}+p^{\prime }$$

where $p_{H}^{\prime }$, $p_{O}^{\prime }$, $p_{C1}^{\prime }$, $p_{\text{OD}}^{\prime }$, $p_{C1D}^{\prime }$ and *p*^′^ are parameters of the QSPR model.

### Descriptors and QSPR models for carboxylic acids

The descriptors were again atomic charges and, similarly as for phenols, two types of QSPR models were developed and evaluated. Specifically, QSPR models with four descriptors (4d QSPR models) and QSPR models with seven descriptors (7d QSPR models). The 4d QSPR models used similar descriptors as the 3d models for phenols - the atomic charge of the hydrogen atom from the COOH group (*q*_*H*_), the charge on the hydrogen bound oxygen atom from the COOH group (*q*_*O*_), and the charge on the carbon atom binding the COOH group (*q*_*C*1_). Additionally, also the charge of the second carboxyl oxygen (*q*_*O*2_) is included. These 4d QSPR models are represented by the equation:

4 $$\;pK_{a}=p_{H}\cdot q_{H}+p_{O}\cdot q_{O}+p_{O2}\cdot q_{O2}+p_{C1}\cdot q_{C1}+p$$

where *p*_*H*_, *p*_*O*_, *p*_*O*2_, *p*_*C*1_ and *p* are parameters of the QSPR model. The 7d QSPR models employ also charges from the dissociated forms, namely the charge on the carboxyl oxygens (*q*_*OD*_, *q*_*O*2*D*_) and the charge on the carboxylic carbon atom (*q*_*C*1*D*_). The equation of the 7d QSPR models is therefore:

5 $$\begin{array}{l} pK_{a}=p_{H}^{\prime }\cdot q_{H}+p_{O}^{\prime }\cdot q_{O}+p_{O2}^{\prime }\cdot q_{O2}+p_{C1}^{\prime }\cdot q_{C1}\\ \;+p_{\text{OD}}^{\prime }\cdot q_{\text{OD}}+p_{O2D}^{\prime }\cdot q_{O2D}+p_{C1D}^{\prime }\cdot q_{C1D}+p^{\prime } \end{array}$$

where $p_{H}^{\prime }$, $p_{O}^{\prime }$, $p_{O2}^{\prime }$, $p_{C1}^{\prime }$, $p_{\text{OD}}^{\prime }$, $p_{O2D}^{\prime }$, $p_{C1D}^{\prime }$ and *p*^′^ are parameters of the QSPR model.

### QSPR model parameterization

The parameterization of the QSPR models was done by multiple linear regression (MLR) using the software tool QSPR Designer [62].

## Results and discussion

### QM and EEM QSPR models for phenols

We prepared one 3d QSPR model and one 5d QSPR model using atomic charges calculated by each of the above mentioned 18 EEM parameter sets. These models are denoted 3d or 5d EEM QSPR models. Additionally, we created one 3d and one 5d QSPR model using atomic charges calculated by each of the corresponding 8 QM charge calculation approaches (denoted as 3d or 5d QM QSPR models). The data set of 74 phenol molecules was used for the parameterization of the QSPR models, and the obtained models were validated for all molecules in the data set.

The parameterization of the 3d EEM QSPR models showed that several molecules in the data set perform as outliers. For this reason, we created also EEM QSPR models without outliers (i.e., EEM QSPR models for which parameterization was done using a data set that excluded the previously observed outliers). These models are denoted 3d EEM QSPR WO models. We classified as outliers 10% of the molecules from our data set, which had the highest Cook’s square distance. Therefore the 3d EEM QSPR WO models were parameterized using 67 molecules, and their validation was also done on the data set excluding outliers.

The quality of the QSPR models, i.e. the correlation between experimental p *K*_*a*_and the p *K*_*a*_calculated by each model, was evaluated using the squared Pearson correlation coefficient (*R*^2^), root mean square error (RMSE), and average absolute p *K*_*a*_error ($\bar{\Delta }$), while the statistical criteria were the standard deviation of the estimation (*s*) and Fisher’s statistics of the regression (*F*).

**Figure 1:**
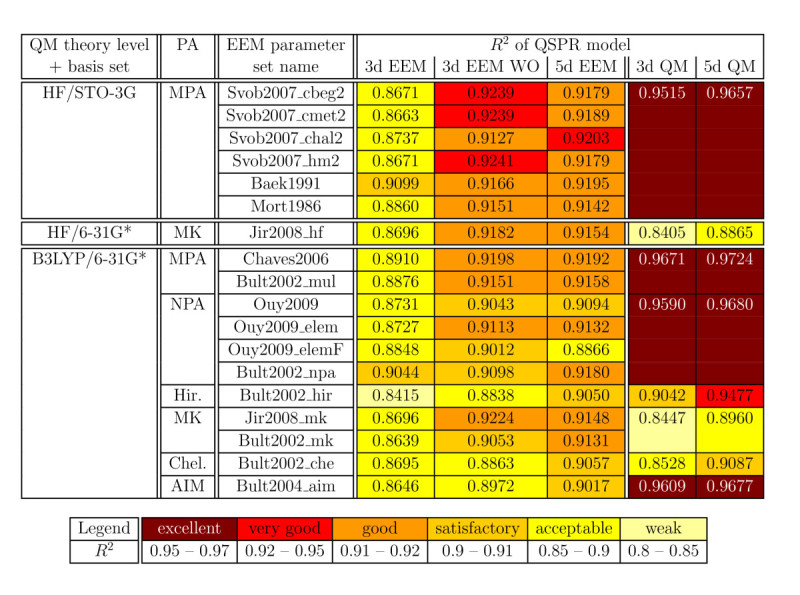
***R*** ^***2***^ **for the correlation between calculated and experimental p** ***K***_***a***_ **.**

**Figure 2:**
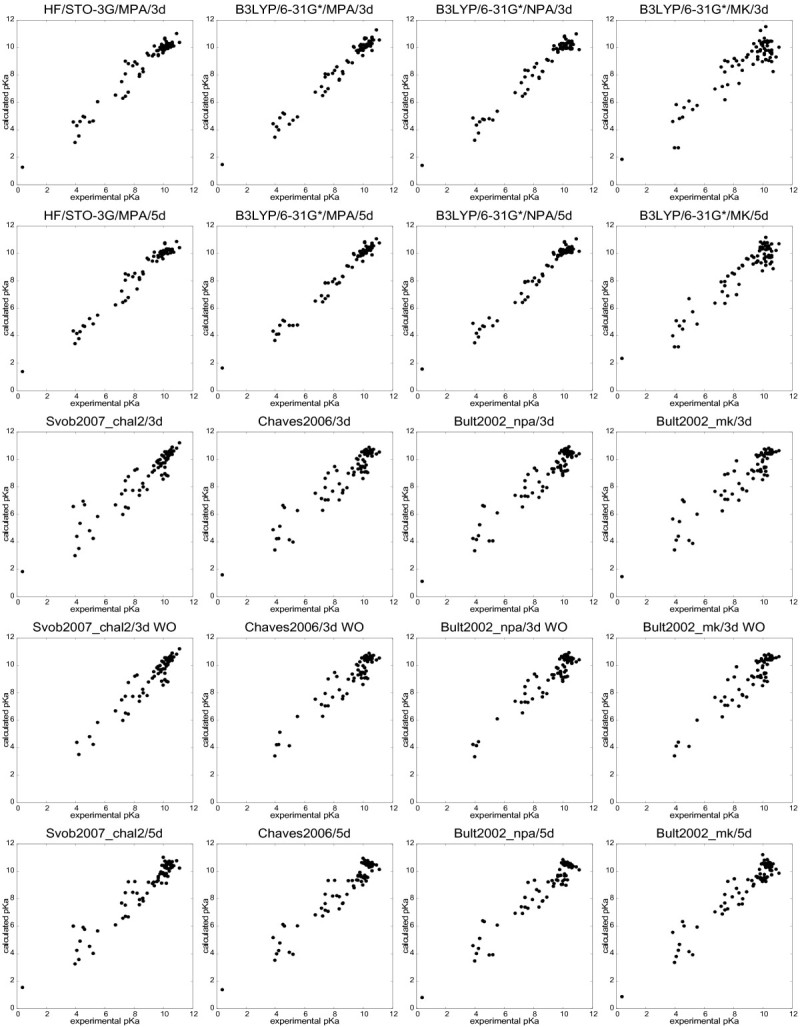
**Correlation graphs.** Graphs showing the correlation between experimental and calculated p *K*_*a*_for selected QSPR models.

Table 2 contains the quality criteria (*R*^2^, RMSE, $\bar{\Delta }$) and statistical criteria (*s* and *F*) for all the QSPR models analyzed. All these models are statistically significant at *p* = 0.01. Since our data sets contained 74 and 67 molecules, respectively, the appropriate *F* value to consider was that for 60 samples. Thus, the 3d QSPR models are statistically significant (at *p*= 0.01) when *F* > 4.126 and the 5d QSPR models when *F* > 3.339. Figure 1 summarizes the *R*^2^ of all QSPR models for ease of visual comparison, and Tables 3 and 4 provide a comparison of the models from specific points of view. The parameters of the QSPR models are summarized in the (Additional file 4: Table S2) and all charge descriptors and p *K*_*a*_values are contained in the (Additional file 5: Table S6). The most relevant graphs of correlation between experimental and calculated p *K*_*a*_are visualized in Figure 2.

**Table 2:** **Quality criteria and statistical criteria for all the QSPR models analyzed in the present study and focused on phenols**

QM theory level	PA	EEM parameter	QSPR model	***R*** ^2^	RMSE	$\bar{\Delta }$	***s***	***F***
+ basis set		set name						
HF/STO-3G	MPA	-	3d QM	0.9515	0.490	0.388	0.504	458
		-	5d QM	0.9657	0.412	0.310	0.430	358
		Svob2007_cbeg2	3d EEM	0.8671	0.812	0.571	0.835	152
			3d EEM WO	0.9239	0.482	0.382	0.497	255
			5d EEM	0.9179	0.638	0.481	0.666	152
		Svob2007_cmet2	3d EEM	0.8663	0.814	0.577	0.837	151
			3d EEM WO	0.9239	0.482	0.386	0.497	255
			5d EEM	0.9189	0.634	0.476	0.661	154
		Svob2007_chal2	3d EEM	0.8737	0.792	0.554	0.814	161
			3d EEM WO	0.9127	0.483	0.387	0.498	220
			5d EEM	0.9203	0.629	0.473	0.656	157
		Svob2007_hm2	3d EEM	0.8671	0.812	0.578	0.835	152
			3d EEM WO	0.9241	0.481	0.387	0.496	256
			5d EEM	0.9179	0.638	0.478	0.666	152
		Baek1991	3d EEM	0.9099	0.669	0.531	0.688	236
			3d EEM WO	0.9166	0.531	0.423	0.548	231
			5d EEM	0.9195	0.632	0.493	0.659	155
		Mort1986	3d EEM	0.8860	0.752	0.577	0.773	181
			3d EEM WO	0.9151	0.520	0.405	0.536	226
			5d EEM	0.9142	0.652	0.524	0.680	145
HF/6–31G*	MK	-	3d QM	0.8405	0.890	0.727	0.915	123
		-	5d QM	0.8865	0.750	0.641	0.782	106
		Jir2008_hf	3d EEM	0.8612	0.830	0.582	0.853	145
			3d EEM WO	0.9182	0.500	0.394	0.516	236
			5d EEM	0.9154	0.648	0.488	0.676	147
B3LYP/6–31G*	MPA	-	3d QM	0.9671	0.404	0.317	0.415	686
		-	5d QM	0.9724	0.370	0.274	0.386	479
		Chaves2006	3d EEM	0.891	0.735	0.570	0.756	191
			3d EEM WO	0.9198	0.505	0.398	0.521	241
			5d EEM	0.9192	0.633	0.489	0.660	155
		Bult2002_mul	3d EEM	0.8876	0.747	0.589	0.768	184
			3d EEM WO	0.9151	0.520	0.416	0.536	226
			5d EEM	0.9158	0.646	0.504	0.674	148
B3LYP/6–31G*	NPA	-	3d QM	0.9590	0.451	0.349	0.464	546
		-	5d QM	0.9680	0.399	0.295	0.416	411
		Ouy2009	3d EEM	0.8731	0.793	0.541	0.815	161
			3d EEM WO	0.9043	0.505	0.379	0.521	198
			5d EEM	0.9094	0.670	0.503	0.699	137
		Ouy2009_elem	3d EEM	0.8727	0.795	0.546	0.817	160
			3d EEM WO	0.9113	0.487	0.382	0.502	216
			5d EEM	0.9132	0.656	0.495	0.684	143
		Ouy2009_elemF	3d EEM	0.8848	0.756	0.519	0.777	179
			3d EEM WO	0.9012	0.512	0.386	0.528	192
			5d EEM	0.8866	0.750	0.520	0.782	106
		Bult2002_npa	3d EEM	0.9044	0.689	0.532	0.708	221
			3d EEM WO	0.9098	0.523	0.405	0.539	212
			5d EEM	0.9180	0.638	0.488	0.666	152
	Hir.	-	3d QM	0.9042	0.689	0.503	0.708	220
		-	5d QM	0.9477	0.509	0.356	0.531	246
		Bult2002_hir	3d EEM	0.8415	0.887	0.636	0.912	124
			3d EEM WO	0.8838	0.579	0.414	0.597	160
			5d EEM	0.9050	0.687	0.522	0.717	130
	MK	-	3d QM	0.8447	0.878	0.705	0.903	127
		-	5d QM	0.8960	0.718	0.594	0.749	117
		Jir2008_dft	3d EEM	0.8696	0.804	0.555	0.827	156
			3d EEM WO	0.9224	0.487	0.371	0.502	250
			5d EEM	0.9148	0.650	0.489	0.678	146
		Bult2002_mk	3d EEM	0.8639	0.822	0.610	0.845	148
			3d EEM WO	0.9053	0.519	0.384	0.535	201
			5d EEM	0.9131	0.657	0.508	0.685	143
	Chel.	-	3d QM	0.8528	0.854	0.712	0.878	135
		-	5d QM	0.9087	0.673	0.552	0.702	135
		Bult2002_che	3d EEM	0.8695	0.805	0.597	0.828	155
			3d EEM WO	0.8863	0.588	0.436	0.606	164
			5d EEM	0.9057	0.684	0.540	0.714	131
	AIM	-	3d QM	0.9609	0.440	0.332	0.452	573
		-	5d QM	0.9677	0.400	0.285	0.417	407
		Bult2004_aim	3d EEM	0.8646	0.819	0.619	0.842	149
			3d EEM WO	0.8972	0.590	0.438	0.608	183
			5d EEM	0.9017	0.698	0.571	0.728	125

**Table 3:** **Average** ***R*** ^***2***^ **between experimental and predicted p** ***K***_***a***_ **for all QSPR models of a certain type and percentages of QSPR models whose** ***R*** ^***2***^ **values are in a certain interval**

QSPR model		3d EEM	3d EEM WO	5d EEM	3d QM	5d QM
Average *R*^2^		0.876	0.911	0.913	0.929	0.951
Interval of *R*^2^	*R*^2^ > 0.9	11%	83%	94%	78%	83%
	0.9 ≥ *R*^2^ > 0.85	83%	17%	6%	6%	17%
	0.85 ≥ *R*^2^ > 0.8	6%	0%	0%	17%	0%
**QSPR model**		**EEM based models**			**QM based models**	
Average *R*^2^		0.900			0.940	
Interval of *R*^2^	*R*^2^ > 0.9	63%			81%	
	0.9 ≥ *R*^2^ > 0.85	35%			13%	
	0.85 ≥ *R*^2^ > 0.8	2%			6%	

**Table 4:** **Average** ***R*** ^***2***^ **between experimental and predicted p** ***K***_***a***_ **for all QSPR models using atomic charges calculated by a specific combination of theory level and basis set, or by a specific population analysis**

QSPR model		3d EEM	3d EEM WO	5d EEM	3d QM	5d QM
Theory level	HF/STO-3G	0.878	0.919	0.918	0.952	0.966
and basis set *	B3LYP/6–31G*	0.889	0.917	0.918	0.967	0.972
Population	MPA	0.889	0.917	0.918	0.967	0.972
analysis **	NPA	0.884	0.907	0.907	0.959	0.968
	Hirshfeld	0.842	0.884	0.905	0.904	0.948
	MK	0.867	0.914	0.914	0.845	0.896
	CHELPG	0.870	0.886	0.906	0.853	0.909
	AIM	0.865	0.897	0.902	0.961	0.968

^*^Only QSPR models employing MPA were included in this analysis.

^**^Only QSPR models using B3LYP/6–31G* were included in this analysis.

### Prediction of p ***K***_***a***_using EEM charges

The key question we wanted to answer in this paper is whether EEM QSPR models are applicable for p *K*_*a*_prediction. For this purpose we selected a set of phenol molecules and generated QSPR models which used EEM atomic charges as descriptors. We then evaluated the accuracy of these models by comparing the predicted p *K*_*a*_values with the experimental ones. The results (see Tables 2 and 3, Figure 1) clearly show that QSPR models based on EEM charges are indeed able to predict the p *K*_*a*_of phenols with very good accuracy. Namely, 63% of the EEM QSPR models evaluated in this study were able to predict p *K*_*a*_with *R*^2^ > 0.9. The average *R*^2^ for all 54 EEM QSPR models considered was 0.9, while the best EEM QSPR model reached *R*^2^ = 0.924. Our findings thus suggest that EEM atomic charges may prove as efficient QSPR descriptors for p *K*_*a*_prediction. The only drawback of EEM is that EEM parameters are currently not available for some types of atoms. Nevertheless, EEM parameterization is still a topic of research, therefore more general parameter sets are being developed.

### Improvement of EEM QSPR models by removing outliers

The quality of 3d EEM QSPR models can be markedly increased by removing the outliers. In this case, the models have average *R*^2^ = 0.911 and 83% of them have *R*^2^ > 0.9. The disadvantage of these models is that they are not able to cover the complete data set (i.e., 10% of molecules must be excluded as outliers).

On the other hand, the outliers are similar for all EEM QSPR models. For example, while 16 molecules from our data set are outliers for at least one parameter set, 10 out of these 16 molecules are outliers for five or more parameter sets. From the chemical point of view, most of the outliers contain one or more nitro groups. This may be related to reported lower accuracy of EEM for these groups [48]. In general one limitation of the 3d EEM QSPR models is that they are very sensitive to the quality of EEM charges. Therefore, if the EEM charges are inaccurate for certain compounds or class of compounds, the 3d QSPR models based on these EEM charges will have lower performance for these compounds or class of compounds. In addition, a lower experimental accuracy of these p *K*_*a*_values may also be a reason for low performance in some cases. A table containing information about outlier molecules is given in the (Additional file 6: Table S3).

### Improvement of EEM QSPR models by adding descriptors

Our first EEM QSPR models contained three descriptors (3d), namely atomic charges originating from the non-dissociated molecule. Nonetheless, in our study we found that using two additional charge descriptors from the dissociated molecule can markedly improve the predictive power of the EEM QSPR models. Tables 2 and 3, Figure 1 show that these new 5d EEM QSPR models provide better p *K*_*a*_prediction than their corresponding 3d EEM QSPR models. Specifically, adding the descriptors derived from the dissociated molecules increased the average *R*^2^ value for the EEM QSPR models from 0.876 to 0.913.

### Comparison of EEM QSPR models and QM QSPR models

Another important question is how accurate the EEM QSPR models are in comparison with QM QSPR models. Table 2 and Figure 1 show that QM QSPR models provide, in most cases, more precise predictions. This is confirmed also by the average *R*^2^ values from Table 3. This is not surprising, since EEM is an empirical method which just mimics the QM approach for which it was parameterized. An interesting fact is that the differences in accuracy between QM QSPR models and EEM QSPR models are not substantial. For example, 5d EEM QSPR models have average *R*^2^ = 0.913, while 5d QM QSPR models have average *R*^2^ = 0.951. We also note that adding more descriptors to a QM QSPR model brings less improvement than adding more descriptors to an EEM QSPR model.

### Influence of theory level and basis set

EEM parameters are available only for a relatively small number of theory levels (HF and B3LYP) and basis sets (STO-3G and 6–31G*). Therefore we can not perform such a deep analysis of theory level and basis set influence on p *K*_*a*_prediction capability from EEM atomic charges, as was done for QM QSPR models by Gross et al. [22] or Svobodova et al. [24]. We can only compare the models employing HF/STO-3G and B3LYP/6–31G* charges, as these are the only combinations for which EEM parameters are available for the same population analysis (MPA). Therefore we can study only the influence of the combination of theory level / basis set, and not the isolated influence of the theory level or basis set. Our analysis revealed that B3LYP/6–31G* charges provide slightly more accurate QM QSPR models than HF/STO-3G charges (see Table 4). This is in agreement with our previous findings [24], and it can be explained by the fact that 6–31G* is a more robust basis set than STO-3G. However, the difference is not marked in the case of EEM QSPR models.

### Influence of population analysis

Eleven EEM parameter sets were published for B3LYP/6–31G* with six different population analyses (see Table 1). Therefore it is straightforward to analyze the influence of the population analysis on the predictive power of the resulting QSPR models (see Table 4). We found that MPA and NPA provide the best QM models, while MK and CHELPG (PAs based on fitting the atomic charges to the molecular electrostatic potential) provide weak QM models. Our results are in agreement with previous studies [22, 24]. QM QSPR models based on AIM predict p *K*_*a*_ with accuracy comparable to MPA and NPA. In the case of EEM QSPR models, we did indeed find that MPA provided the best models, but most of the other population analyses gave comparable results. This confirms previous observations that the EEM approach is not able to faithfully mimic MK charges [63]. On the other hand, this drawback of EEM allowed the EEM QSPR models employing MK charges to predict p *K*_*a*_more accurately than the corresponding QM QSPR models.

### Influence of the EEM parameter set

Two or more EEM parameter sets are available in literature for four combinations of theory level, basis set and population analysis (see Table 1). We found that the quality of EEM QSPR models employing the same types of charges slightly varies when using EEM parameters coming from different studies (see Table 2 and Figure 1). Even EEM parameters from the same study, but obtained by different approaches, lead to QSPR models of slightly different quality. In any case, these differences are minimal.

### Comparison with previous work

QM QSPR models for p *K*_*a*_prediction in phenols, similar to those presented in this paper (i.e., employing similar charges) were previously published by Gross and Seybold [22], Kreye and Seybold [23] and Svobodova and Geidl [24]. Table 5 shows a comparison between these models and the models developed in this study. Our work is the first which presents QSPR models for p *K*_*a*_prediction based on EEM charges. Therefore, we can not provide a comparison between EEM QSPR models, but we can compare against QSPR models based on QM charges only. It is seen therein that our 3d QM QSPR models show markedly higher *R*^2^ and *F* values than the models published by Gross and Seybold and Kreye and Seybold (even if some of these models employ higher basis sets) and comparable *R*^2^ and *F* values as models published by Svobodova and Geidl. Moreover, our 5d QM QSPR models outperform the models from Svobodova and Geidl. Our best EEM QSPR models (i.e., 5d EEM QSPR models) provide even better results than QM QSPR models from Gross and Seybold and Kreye and Seybold. These EEM QSPR models are not as accurate as the QM QSPR models published by Svobodova and Geidl or those developed in this work, but the loss of accuracy is not too high (*R*^2^ values are still > 0.91).

**Table 5:** **Comparison between the performance of the QSPR models developed here, and previously developed models**

	Theory							Number of	
Method	level	PA	Basis set	Descriptors	***R*** ^2^	***s***	***F***	molecules	Source
QM	B3LYP	NPA	6–311G**	*q*_*OH*_	0.789	1.300	48	15	Kreye and Seybold [23]^*a*^
	B3LYP	NPA	6–311G**	*q*_*O*_	0.731	1.500	38	15	Kreye and Seybold [23]^*a*^
	B3LYP	NPA	6–31+G*	*q*_*OH*_	0.880	0.970	95	15	Kreye and Seybold [23]^*b*^
	B3LYP	NPA	6–31+G*	*q*_*O*_	0.865	1.000	38	15	Kreye and Seybold [23]^*b*^
	B3LYP	NPA	6–311G(d,p)	$q_{O^{-}}$	0.911	0.252	173	19	Gross and Seybold [22]
	B3LYP	NPA	6–311G(d,p)	*q*_*H*_	0.887	0.283	134	19	Gross and Seybold [22]
	B3LYP	NPA	6–31G*	*q*_*H*_, *q*_*O*_, *q*_*C*1_	0.961	0.440	986	124	Svobodova and Geidl [24]
	B3LYP	NPA	6–311G	*q*_*H*_, *q*_*O*_, *q*_*C*1_	0.962	0.435	1013	124	Svobodova and Geidl [24]
	B3LYP	NPA	6–31G*	*q*_*H*_, *q*_*O*_, *q*_*C*1_	0.959	0.464	545	74	This work
	B3LYP	NPA	6–31G*	*q*_*H*_, *q*_*O*_, *q*_*C*1_,	0.968	0.410	705	74	This work
				*q*_*OD*_, *q*_*C*1*D*_					
EEM	B3LYP	NPA	6–31G*	*q*_*H*_, *q*_*O*_, *q*_*C*1_,	0.918	0.656	261	74	This work ^*c*^
				*q*_*OD*_, *q*_*C*1*D*_					
QM	B3LYP	MPA	6–311G(d,p)	*q*_*H*_	0.913	0.248	179	19	Gross and Seybold [22]
	B3LYP	MPA	6–311G(d,p)	$q_{O^{-}}$	0.894	0.274	144	19	Gross and Seybold [22]
	B3LYP	MPA	6–311G	*q*_*H*_, *q*_*O*_, *q*_*C*1_	0.938	0.556	605	124	Svobodova and Geidl [24]
	B3LYP	MPA	6–31G*	*q*_*H*_, *q*_*O*_, *q*_*C*1_	0.959	0.450	936	124	Svobodova and Geidl [24]
	B3LYP	MPA	6–31G*	*q*_*H*_, *q*_*O*_, *q*_*C*1_	0.967	0.415	685	74	This work
	B3LYP	MPA	6–31G*	*q*_*H*_, *q*_*O*_, *q*_*C*1_,	0.972	0.380	822	74	This work
				*q*_*OD*_, *q*_*C*1*D*_					
EEM	B3LYP	MPA	6–31G*	*q*_*H*_, *q*_*O*_, *q*_*C*1_,	0.919	0.651	265	74	This work^*d*^
				*q*_*OD*_, *q*_*C*1*D*_					
QM	B3LYP	MK	6–311G(d,p)	*q*_*H*_	0.344	0.682	9	19	Gross and Seybold [22]
	B3LYP	MK	6–311G(d,p)	$q_{O^{-}}$	0.692	0.467	38	19	Gross and Seybold [22]
	B3LYP	MK	6–311G	*q*_*H*_, *q*_*O*_, *q*_*C*1_	0.822	0.941	185	124	Svobodova and Geidl [24]
	B3LYP	MK	6–31G*	*q*_*H*_, *q*_*O*_, *q*_*C*1_	0.808	0.978	168	124	Svobodova and Geidl [24]
	B3LYP	MK	6–31G*	*q*_*H*_, *q*_*O*_, *q*_*C*1_	0.845	0.902	126	74	This work
	B3LYP	MK	6–31G*	*q*_*H*_, *q*_*O*_, *q*_*C*1_	0.896	0.739	201	74	This work
				*q*_*OD*_, *q*_*C*1*D*_					
EEM	B3LYP	MK	6–31G*	*q*_*H*_, *q*_*O*_, *q*_*C*1_	0.915	0.669	250	74	This work^*e*^
				*q*_*OD*_, *q*_*C*1*D*_					

^a^With solvent model SM5.4.

^b^With solvent model SM8.

^c^EEM parameter set Bult2002 npa.

^d^EEM parameter set Chaves2006.

^e^EEM parameter set Jir2008 mk.

### Cross-validation

Our results show that 5d EEM QSPR models provide a fast and accurate approach for p *K*_*a*_prediction. Nonetheless, the robustness of these models should be proved. Therefore, all the 5d EEM QSPR models (i.e., 18 models) were tested by cross-validation. For comparison, also the cross-validation of all 5d QM QSPR models (i.e., 8 models) was done. The *k*-fold cross-validation procedure was used [64, 65], where *k* = 5. Specifically, the set of phenol molecules was divided into five parts (each contained 20% of the molecules). The division was done randomly, and included stratification by p *K*_*a*_value. Afterwards, five cross validation steps were performed. In the first step, the first part was selected as a test set, and the remaining four parts were taken together as the training set. The test and training sets for the other steps were prepared in a similar manner, by subsequently considering one part as a test set, while the remaining parts served as a training set. For each step, the QSPR model was parameterized on the training set. Afterwards, the p *K*_*a*_values of the respective test molecules were calculated via this model, and compared with experimental p *K*_*a*_values. The results are summarized in the (Additional file 7: Table S4), while the cross-validation results for the best and the worst performing 5d EEM QSPR models are shown in Table 6. The cross-validation showed that the models are stable and the values of *R*^2^ and RMSE are similar for the test set, the training set and the complete set. The robustness of EEM QSPR models and QM QSPR models is comparable.

**Table 6:** **Comparison of the quality criteria and statistical criteria for the training set, test set and complete set for some selected charge calculation approaches**

5d EEM QSPR model employing Svob2007_chal2 EEM parameters:										
Complete set:										
*R* ^2^	RMSE	*s*	*F*	Number of molecules						
0.920	0.629	0.647	269	74						
**Cross-validation:**										
Cross-	Training set					Test set				
validation					Number of					Number of
step	*R* ^2^	RMSE	*s*	*F*	molecules	*R* ^2^	RMSE	*s*	*F*	molecules
1	0.9283	0.5211	0.5498	137	59	0.9202	1.0754	1.3884	21	15
2	0.9210	0.6538	0.6899	124	59	0.9029	0.5394	0.6963	17	15
3	0.9191	0.6442	0.6796	120	59	0.9275	0.5823	0.7517	23	15
4	0.9207	0.6244	0.6588	123	59	0.9271	0.6878	0.8880	23	15
5	0.9274	0.6302	0.6643	138	60	0.9008	0.6678	0.8834	15	14
**5d EEM QSPR model employing Ouy2009_elemF EEM parameters:**										
**Complete set:**										
*R* ^2^	RMSE	*s*	*F*	Number of molecules						
0.8866	0.7501	0.7825	106	74						
**Cross-validation:**										
Cross-	Training set					Test set				
validation					Number of					Number of
step	*R* ^2^	RMSE	*s*	*F*	molecules	*R* ^2^	RMSE	*s*	*F*	molecules
1	0.8936	0.6349	0.6698	89	59	0.8704	1.2857	1.6598	12	15
2	0.8953	0.7526	0.7940	91	59	0.8018	0.7802	1.0072	7	15
3	0.8908	0.7481	0.7893	86	59	0.8647	0.7983	1.0306	12	15
4	0.8821	0.7614	0.8033	79	59	0.9154	0.7481	0.9658	19	15
5	0.8956	0.7557	0.7966	93	60	0.8089	0.8396	1.1107	7	14

### Case study for carboxylic acids

**Figure 3:**
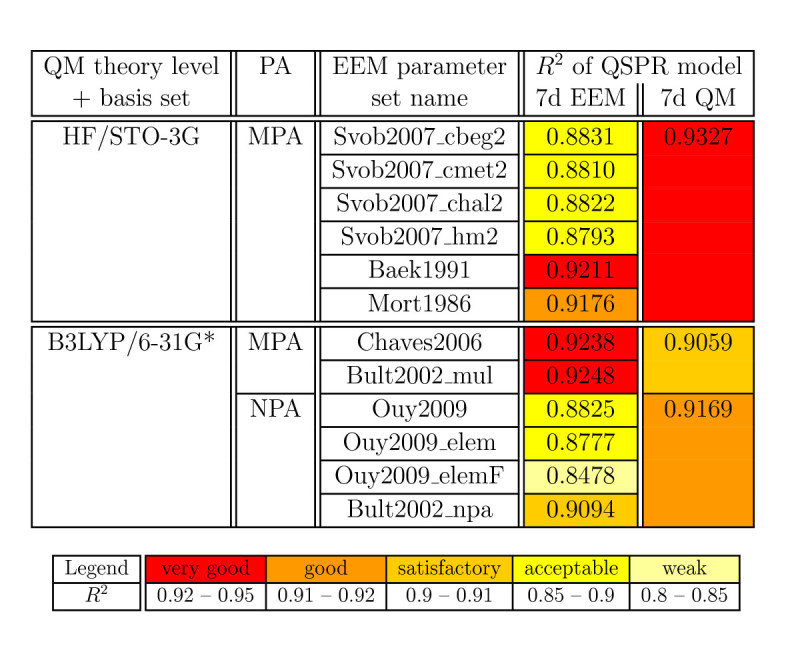
**Correlation between calculated and experimental p** ***K***_***a***_ **for carboxylic acids**

We have shown that QSPR models based on EEM atomic charges can be used for predicting p *K*_*a*_in phenols. In order to evaluate the general applicability of this approach for p *K*_*a*_prediction, we tested the performance of such models for carboxylic acids. This case study is done for the charge schemes found to provide the best QM and EEM QSPR models in the case of phenols. Specifically, QM charges calculated by HF/STO-3G/MPA, B3LYP/6–31G*/MPA and B3LYP/6–31G*/NPA, and EEM charges calculated using the corresponding EEM parameters. Because 5d QSPR models provide the most accurate prediction for phenols, the case study is focused on their analogue for carboxylic acids, i.e., 7d QSPR models. Squared Pearson correlation coefficients of the analysed QSPR models are summarized in Figure 3, and all the quality and statistical criteria can be found in (Additional file 8: Table S5). The results show that 7d EEM QSPR models are able to predict the p *K*_*a*_of carboxylic acids with very good accuracy. Namely, 5 out of 12 analysed 7d EEM QSPR models were able to predict p *K*_*a*_with *R*^2^ > 0.9, while the best EEM QSPR model reached *R*^2^ = 0.925. Therefore, we concluded that EEM QSPR models are indeed applicable also for carboxylic acids. Again QM QSPR models perform better than EEM QSPR models, but the differences are not substantial.

## Conclusions

We found that the QSPR models employing EEM charges can be a suitable approach for p *K*_*a*_prediction. From our 54 EEM QSPR models focused on phenols, 63% show a correlation of *R*^2^ > 0.9 between the experimental and predicted p *K*_*a*_. The most successful type of these EEM QSPR models employed 5 descriptors, namely the atomic charge of the hydrogen atom from the phenolic OH group, the charge on the oxygen atom from the phenolic OH group, the charge on the carbon atom binding the phenolic OH group, the charge on the oxygen from the phenoxide O^-^ from the dissociated molecule, and the charge on the carbon atom binding this oxygen. Specifically, 94% of these models have *R*^2^ > 0.9, and the best one has *R*^2^ = 0.920. In general, including charge descriptors from dissociated molecules, which was introduced in our work, always increases the quality of a QSPR model. The only drawback of EEM QSPR models is that the EEM parameters are currently not available for all types of atoms. Therefore the EEM parameter sets need to be expanded to larger sets of molecules and further improved.

As expected, the QM QSPR models provided more accurate p *K*_*a*_predictions than the EEM QSPR models. Nevertheless, these differences are not substantial. Furthermore, a big advantage of EEM QSPR models is that one can calculate the EEM charges markedly faster than the QM charges. Moreover, the EEM QSPR models are not so strongly influenced by the charge calculation approach as the QM QSPR models are. Specifically, the QM QSPR models which use atomic charges obtained from calculations with higher basis set perform better, while the EEM QSPR models do not show such marked differences. Similarly, the quality of QM QSPR models depends a lot on population analysis, but EEM QSPR models are not influenced so much. Namely, QM QSPR models which use atomic charges calculated from MPA, NPA and Hirshfeld PA performed very well, while MK provides only weak models. In the case of EEM QSPR models, MPA performs also the best, but all other PAs (including MK) provide accurate results as well. The source of the EEM parameters also did not affect the quality of the EEM QSPR models significantly.

The robustness of EEM QSPR models was successfully confirmed by cross-validation. Specifically, the accuracy of p *K*_*a*_prediction for the test, training and complete set were comparable. The applicability of EEM QSPR models for other chemical classes was tested in a case study focused on carboxylic acids. This case study showed that EEM QSPR models are indeed applicable for p *K*_*a*_prediction also for carboxylic acids. Namely, 5 from 12 of these models were able to predict p *K*_*a*_with *R*^2^ > 0.9, while the best EEM QSPR model reached *R*^2^ = 0.925.

Therefore, EEM QSPR models constitute a very promising approach for the prediction of p *K*_*a*_. Their main advantages are that they are accurate, and can predict p *K*_*a*_values very quickly, since the atomic charge descriptors used in the QSPR model can be obtained much faster by EEM than by QM. Additionally, the quality of EEM QSPR models is less dependent on the type of atomic charges used (theory level, basis set, population analysis) than in the case of QM QSPR models. Accordingly, EEM QSPR models constitute a p *K*_*a*_prediction approach which is very suitable for virtual screening.

## Author’s contributions

The concept of the study originated from JK and was reviewed and extended by RA, while the design was put together by RSV and SG and reviewed by JK and RA. SG and CMI collected and prepared the input data. SG, OS, DS and TB performed the acquisition and post-processing of data. The data were analyzed and interpreted by RSV, SG, CMI and JK. The manuscript was written by RSV and SG in cooperation with JK and CMI, and reviewed by all authors.

## Authors’ information

Radka Svobodová Vařeková and Stanislav Geidl wish it to be known that, in their opinion, the first two authors should be regarded as joint First Authors.

## Electronic supplementary material

Additional file 1: Table S1a: The list of the phenol molecules, including their names, NCS numbers, CAS numbers and experimental p *K*_*a*_values. (XLS 36 KB)

Additional file 2: Molecules. The SDF files with the structures of the molecules and also their dissociated forms. (ZIP 310 KB)

Additional file 3: Table S1b: The list of the carboxylic acid molecules, including their names, NCS numbers, CAS numbers and experimental p *K*_*a*_values. (XLS 36 KB)

Additional file 4: Table S2: The parameters of all the QSPR models for phenols. (XLS 46 KB)

Additional file 5: Table S6: The table containing charge descriptors for all charge calculation approaches and predicted p *K*_*a*_values for all QSPR models (for phenols). (XLS 421 KB)

Additional file 6: Table S3: The information about outlier molecules for phenols. (XLS 46 KB)

Additional file 7: Table S4: The table of cross-validation results for phenols. (XLS 174 KB)

Additional file 8: Table S5: The quality and statistical criteria of QSPR models for carboxylic acids. (XLS 130 KB)

Below are the links to the authors’ original submitted files for images.

Authors’ original file for figure 1

Authors’ original file for figure 2

Authors’ original file for figure 3

## Abbreviations

3d: 3 descriptors
4d: 4 descriptors
5d: 5 descriptors
7d: 7 descriptors
AIM: Atoms in Molecules
ANN: Artificial Neural Networks
B3LYP: Becke, three-parameter, Lee-Yang-Parr
DENR: Dynamic Electronegativity Relaxation
EEM: Electronegativity Equalization Method
GDAC: Geometry-Dependent Atomic Charge
HF: Hartree-Fock
KCM: Kirchhoff Charge Model
LFER: Linear Free Energy Relationships
MK: Merz-Singh-Kollman
MLR: Multiple Linear Regression
MP2: Møller-Plesset Perturbation Theory
MPA: Mulliken Population Analysis
NPA: Natural Population Analysis
PA: Population Analysis
PEOE: Partial Equalization of Orbital Electronegativity
QEq: Charge Equilibration
QM: Quantum Mechanical
QSPR: Quantitative Structure-Property Relationship
RMSE: Root Mean Square Error
SQE: Split Charge Equilibration
TSEF: Topologically Symmetric Energy Function.
