# Supplementary material for: Predicting pKa values from EEM atomic charges
Source: J Cheminform. 2013 Apr 10;5:18. doi: 10.1186/1758-2946-5-18 (PMC3663834; doi:10.1186/1758-2946-5-18)
Supplement: Supplementary file 9 — Authors’ original file for figure 1 [file 13321_2012_461_MOESM9_ESM.pdf]

QM theory level + basis set	PA	EEM parameter set name	R^2 of QSPR model				
			3d EEM	3d EEM WO	5d EEM	3d QM	5d QM
HF/STO-3G	MPA	Svob2007_cbeg2	0.8671	0.9239	0.9179	0.9515	0.9657
		Svob2007_cmet2	0.8663	0.9239	0.9189		
		Svob2007_chal2	0.8737	0.9127	0.9203		
		Svob2007_hm2	0.8671	0.9241	0.9179		
		Baek1991	0.9099	0.9166	0.9195		
		Mort1986	0.8860	0.9151	0.9142		
HF/6-31G*	MK	Jir2008_hf	0.8696	0.9182	0.9154	0.8405	0.8865
B3LYP/6-31G*	MPA	Chaves2006	0.8910	0.9198	0.9192	0.9671	0.9724
		Bult2002_mul	0.8876	0.9151	0.9158		
	NPA	Ouy2009	0.8731	0.9043	0.9094	0.9590	0.9680
		Ouy2009_elem	0.8727	0.9113	0.9132		
		Ouy2009_elemF	0.8848	0.9012	0.8866		
		Bult2002_npa	0.9044	0.9098	0.9180		
	Hir.	Bult2002_hir	0.8415	0.8838	0.9050	0.9042	0.9477
	MK	Jir2008_mk	0.8696	0.9224	0.9148	0.8447	0.8960
		Bult2002_mk	0.8639	0.9053	0.9131		
	Chel.	Bult2002_che	0.8695	0.8863	0.9057	0.8528	0.9087
	AIM	Bult2004_aim	0.8646	0.8972	0.9017	0.9609	0.9677

Legend	excellent	very good	good	satisfactory	acceptable	weak
R^2	0.95 – 0.97	0.92 – 0.95	0.91 – 0.92	0.9 – 0.91	0.85 – 0.9	0.8 – 0.85
