# Supplementary figures and images for: Predicting pKa values from EEM atomic charges
Source: J Cheminform. 2013 Apr 10;5:18. doi: 10.1186/1758-2946-5-18 (PMC3663834; doi:10.1186/1758-2946-5-18)

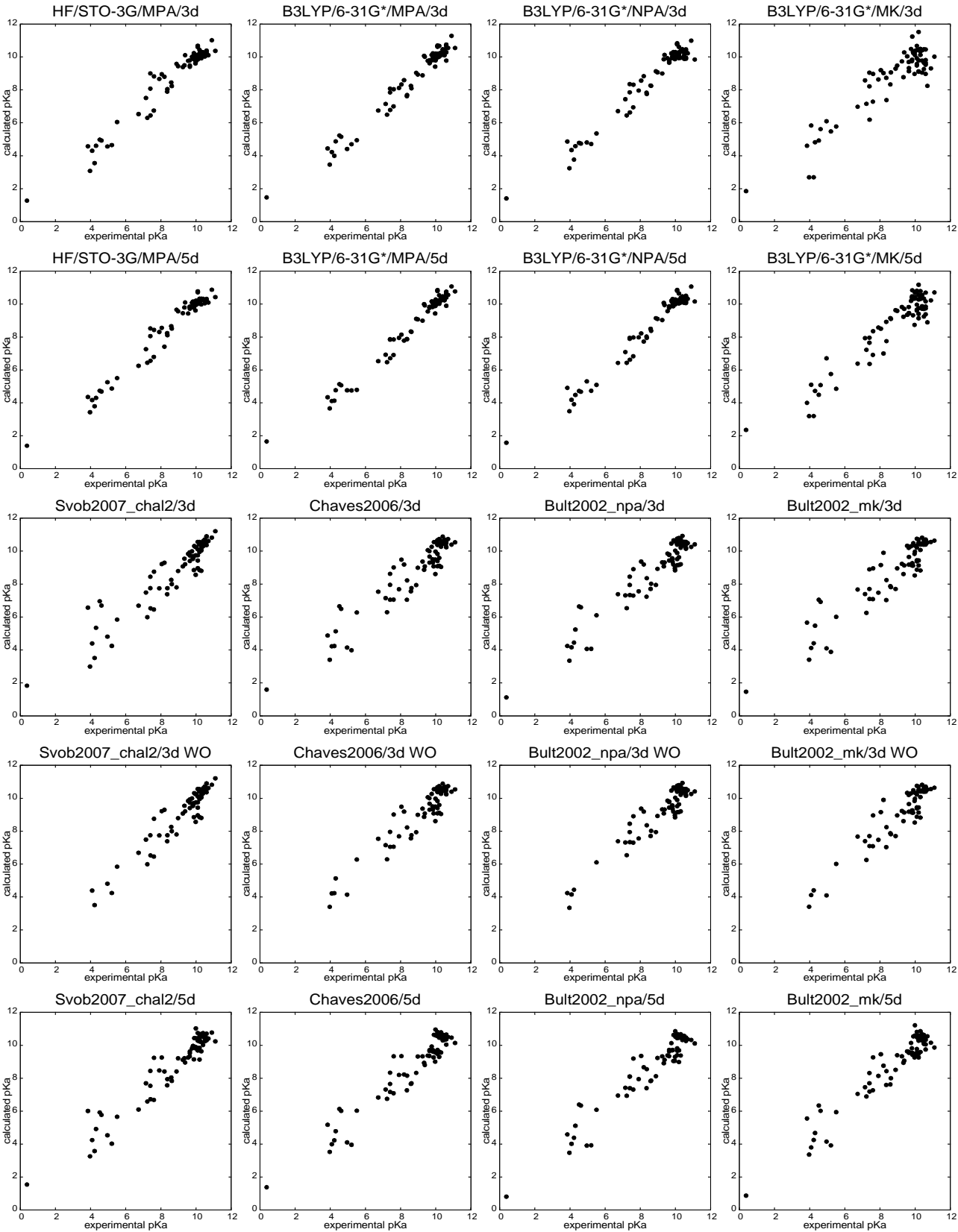

Supplement: Supplementary file 10 — Authors’ original file for figure 2 [file 13321_2012_461_MOESM10_ESM.pdf]
