# Supplementary material for: Predicting pKa values from EEM atomic charges
Source: J Cheminform. 2013 Apr 10;5:18. doi: 10.1186/1758-2946-5-18 (PMC3663834; doi:10.1186/1758-2946-5-18)
Supplement: Supplementary file 11 — Authors’ original file for figure 3 [file 13321_2012_461_MOESM11_ESM.pdf]

QM theory level + basis set	PA	EEM parameter set name	R^2 of QSPR model 7d EEM	R^2 of QSPR model 7d QM
HF/STO-3G	MPA	Svob2007_cbeg2	0.8831	0.9327
		Svob2007_cmet2	0.8810	
		Svob2007_chal2	0.8822	
		Svob2007_hm2	0.8793	
		Baek1991	0.9211	
		Mort1986	0.9176	
B3LYP/6-31G*	MPA	Chaves2006	0.9238	0.9059
		Bult2002_mul	0.9248	
	NPA	Ouy2009	0.8825	0.9169
		Ouy2009_elem	0.8777	
		Ouy2009_elemF	0.8478	
		Bult2002_npa	0.9094	

Legend	very good	good	satisfactory	acceptable	weak
R^2	0.92 – 0.95	0.91 – 0.92	0.9 – 0.91	0.85 – 0.9	0.8 – 0.85
